# The Blood Compatibilities of Blood Purification Membranes and Other Materials Developed in Japan

**DOI:** 10.1155/2011/375390

**Published:** 2011-09-28

**Authors:** Takaya Abe, Karen Kato, Tomoaki Fujioka, Tadao Akizawa

**Affiliations:** ^1^Department of Blood Purification Therapy & Urology, Iwate Medical University, School of Medicine, Iwate 020-8505, Japan; ^2^Division of Nephrology, Department of Medicine, Showa University, School of Medicine, Japan

## Abstract

The biocompatibilities in blood purification therapy are defined as “a concept to stipulate safety of blood purification therapy by an index based on interaction in the body arising from blood purification therapy itself.” The biocompatibilities are associated with not only materials to be used but also many factors such as sterilization method and eluted substance. It is often evaluated based on impacts on cellular pathways and on humoral pathways. Since the biocompatibilities of blood purification therapy in particular hemodialysis are not just a prognostic factor for dialysis patients but a contributory factor for long-term complications, it should be considered with adequate attention. It is important that blood purification therapy should be performed by consistently evaluating not only risks associated with these biocompatibilities but also the other advantages obtained from treatments. In this paper, the biocompatibilities of membrane and adsorption material based on Japanese original which are used for blood purification therapy are described.

## 1. Introduction

The biocompatibilities in blood purification therapy are defined as “a concept to stipulate safety of blood purification therapy by an index based on interaction in the body arising from blood purification therapy itself.” The biocompatibilities are associated with not only materials to be used but also sterilization method, eluted substance, medical agents such as anticoagulant, dialysate solution, and contamination, and so on. It is often evaluated based on impacts on cellular pathways such as leukocyte and platelet as well as on humoral pathways such as complement system, coagulation/fibrinolysis system, kallikrein-kinin system, and cytokine. For hemodialysis the biocompatibilities on blood purification therapy are not just a prognostic factor for dialysis patients but a contributory factor for long-term complications such as immunodeficiency, cardiovascular disease, and dialysis-related amyloidosis (DRA). The material of dialyzer is roughly classified into cellulose type membrane such as cellulose triacetate (CTA) membrane and synthetic type membrane including polyethersulfone (PES) membrane, polymethylmethacrylate (PMMA) membrane, ethylene vinyl alcohol (EVAL) membrane, and vitamin E-coated polysulfone (PS) membrane. 

 In this paper, the biocompatibilities of membrane for dialysis and adsorption material for blood purification therapy which were developed in Japan are described.

## 2. Cellulose-Type Dialyzer

### 2.1. Cellulose Triacetate (CTA) Membrane

**Figure 1:**
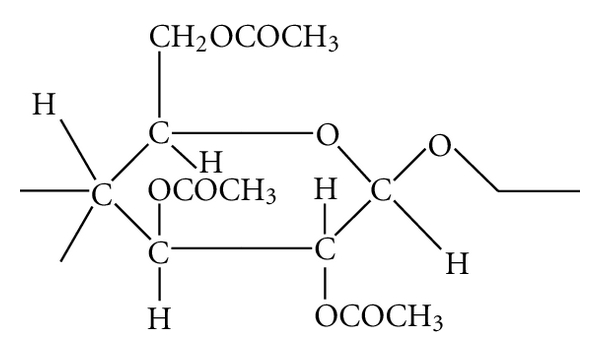
The structural formula of CTA. CTA is formed by substituting three hydroxyls within cellulose.

Cellulose acetate membrane is a kind of cellulose-type membrane which is synthesized by a reaction of natural polymer cellulose and acetic acid, having properties of higher transparency and toughness among thermoplastics. Cellulose diacetate is formed by substituting two hydroxyls within cellulose, and CTA is formed by substituting three hydroxyls (Figure 1). As the number of substitutions increases, hydrophilic property of the membrane decreases and complement activation of the membrane also decreases, as a result biocompatibility of the membrane improves.

**Figure 2:**
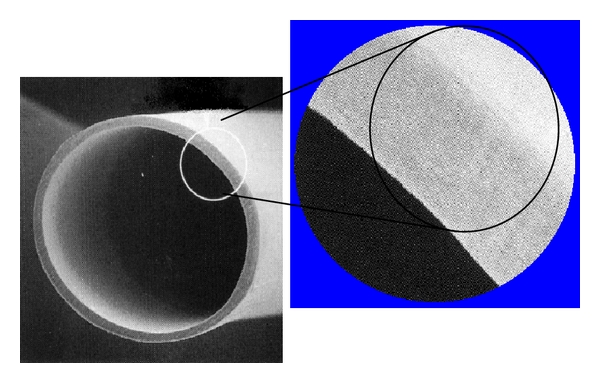
The scanning electron microscopy findings of cross-sectional structure of CTA. The cross-sectional structure of CTA shows uniform structure in which pore size from inside (blood side) to outside (dialysate side) the hollow fiber is equal.

This membrane is characterized by the thickness of 15 *μ*m, the thinnest among commercial dialyzers in Japan, with a uniform cross-sectional structure (Figure 2) in which pore size from inside (blood side) to outside (dialysate side) the hollow fiber is equal. Compared with those of asymmetric membrane, pore size on dialysate side is relatively smaller and that on blood side is relatively larger. Therefore, it has a characteristic that endotoxin-like substances in the dialysate are hard to permeate from dialysate side.

Since hydroxyl group in cellulose is substituted by acetyl group in CTA, complement activation by hydroxyl during dialysis as well as variations in leukocyte and granulocyte elastase is infrequently observed. Hydrophilic property of hemodialysis membrane is prone to activate coagulation factor, whereas its hydrophobic property brings about strong reaction on platelet. Property of CTA has well balance of both hydrophilicity and hydrophobicity, and it has been reported to be excellent in antithrombogenicity [1].

## 3. Synthetic Membrane Dialyzer

Synthetic type membrane is hydrophobic with a property to cause thrombosis and coagulation in contact with blood. Therefore, in case of synthetic membrane, hydrophobic functional group is substituted by hydrophilic group, or acrylic acid or polyvinylpyrrolidone (PVP) is used as a hydrophilizing agent. Possible elution from membrane has been reported since the PVP is hydrosoluble [2].

### 3.1. Polyethersulfone (PES) Membrane

**Figure 3:**
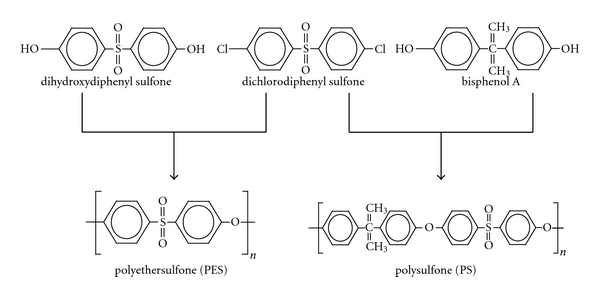
The structural formulas of PES and PS. PS membrane is synthesized by polymerizing dichlorodiphenyl sulfone with bisphenol A, whereas PES is done by polymerizing dichlorodiphenyl sulfone with dihydroxydiphenyl sulfone. Therefore, PES has similar property to PS, but risk of bisphenol A elution from membrane material is lower than that of PS.

PS membrane is synthesized by polymerizing dichlorodiphenyl sulfone with bisphenol A, whereas PES is done by polymerizing dichlorodiphenyl sulfone with dihydroxydiphenyl sulfone (Figure 3). Therefore, PES has a similar property to that of PS, but, it is considered as bisphenol A-free membrane [3]. Further, since PES characteristically has not aliphatic part which exists in PS and atomic weight ratio of sulfone group is higher than that of PS, heat resistance, mechanical resistance, and hydrophilicity are even higher. Moreover, hydrophilicity has been further enhanced by adopting PVP as a hydrophilizing agent in the process of PES membrane manufacturing. In addition, the hydrophilicity based on the PVP exerts antithrombogenicity by inhibiting protein fouling on membrane surface.

**Figure 4:**
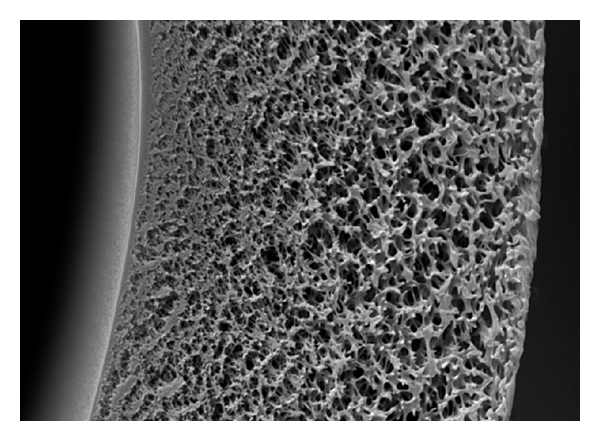
The scanning electron microscopy findings of cross-sectional structure of PES. The cross-section structure of PES has an asymmetric three-layer structure having a compact layer with fine pore size on the inside and outside of the hollow fiber as well as a support layer in the central part thereof.

PES membrane is characterized in the cross-section structure of this membrane (Figure 4). It has an asymmetric three-layer structure having a compact layer with fine pore size on the inside and outside of the hollow fiber as well as a support layer in the central part thereof. With a membrane thickness of 40 *μ*m, the mechanical strength of this membrane depends on its support layer in the central part and the molecule sieving effect of the blood is provided by the compact layer inside the hollow fiber. 

**Figure 5:**
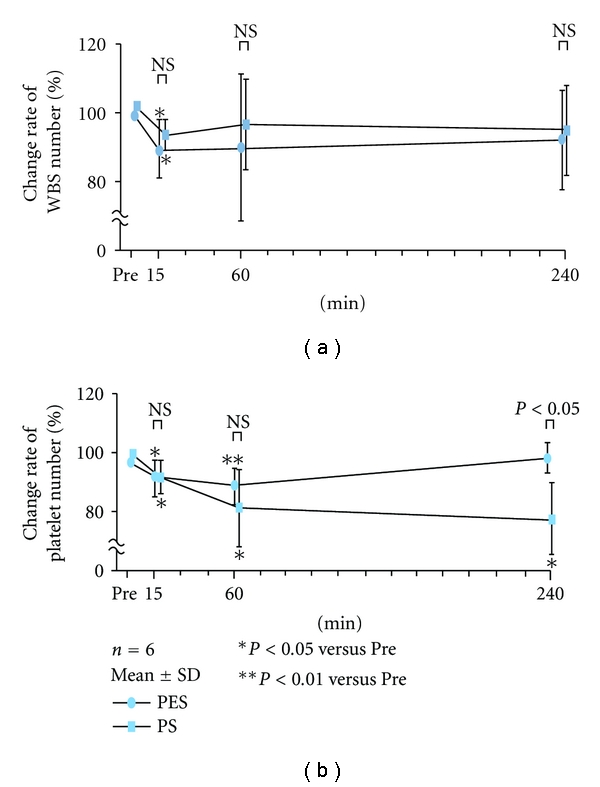
The change of WBC and platelet number during HD session by using PSE-150D (Nipro Co., Ltd., Osaka, Japan) and PS. Compared with PS, PES has excellent biocompatibility, in particular, relating to variation in platelet due to its high hydrophilicity (This figure is due to Dr. Nii's kindness.)

Excellent biocompatibility has been reported for PSE-150D (Nipro Co., Ltd., Osaka, Japan), which adopts PES membrane, without any significant change in leukocyte, platelet, C3a, and granulocyte elastase observed during dialysis therapy [4]. With equivalent or better biocompatibility compared with PS membrane, it works more excellently (Figure 5), in particular, relating to variation in platelet due to its high hydrophilicity. 

### 3.2. Polymethylmethacrylate (PMMA) Membrane

PMMA membrane is produced by mixing and solving isotactic PMMA and syndiotactic PMMA which are different in steric structure. By using PMMA, it is possible to produce membrane with wide range of pore sizes by changing conditions including the blend ratio and also it is possible to produce negatively charged membrane depending on the types of additive agent to be used. It has adsorptive capacity and because of negative membrane charge, in particular, it adsorbs larger amount of basic protein. 

**Figure 6:**
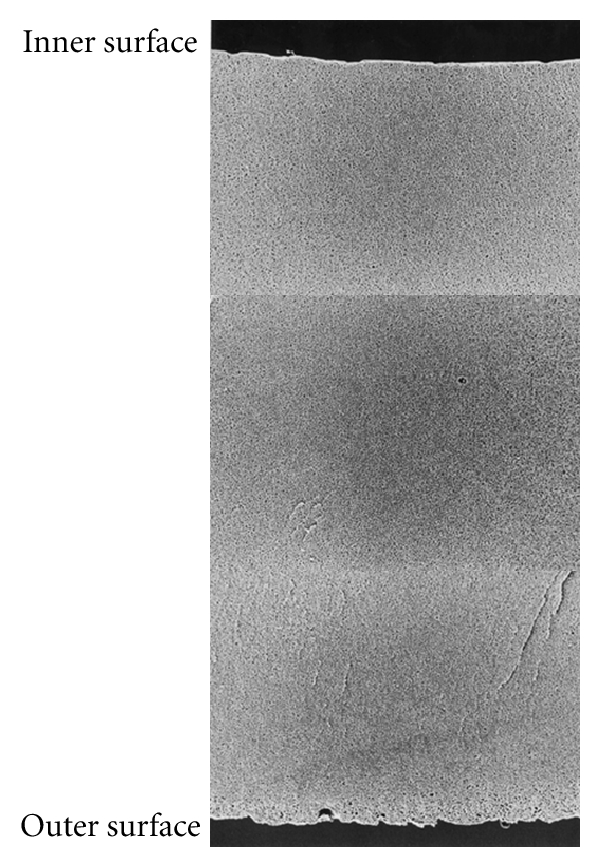
The scanning electron microscopy findings of cross-section structure of PMMA. PMMA has a symmetric structure having nearly homogenous microscopic pores from inner surface to outer surface of hollow fiber.

 PMMA membrane is characterized by the finding that cross-section structure of the membrane (Figure 6) has a symmetric structure having nearly homogenous microscopic pores from inner surface to outer surface of the hollow fiber. The pore sizes on the inner surface of PMMA are larger compared with those of PS membrane. Therefore, PMMA membrane has lower sieving coefficient for beta_2_-microglobulin (*β*2-MG) area than that of PS membrane but it is excellent in absorptive removal of substances with molecular weight of *β*2-MG and those more than 50,000 dalton, which are hard to remove by PS membrane. 

PMMA membrane is known for its excellent biocompatibility based on many reports that it causes less cytokine production such as TNF-*α* and NO synthesis [5]. It has also been reported that PMMA membrane adsorbs and removes factor D which triggers alternative pathway complement activation [6]. In cases of acute kidney injury, it has been reported that PMMA membrane with excellent biocompatibility achieves higher survival rate compared with a case of Cuprophan (regenerated cellulose) membrane [7]. 

### 3.3. Ethylene Vinyl Alcohol (EVAL) Membrane

EVAL membrane is produced by solution polymerization between ethylene and vinyl acetate as well as alkali saponification. As chemical structural formula of EVAL membrane is expressed that vinyl alcohol group with hydrophilicity and ethylene group with hydrophobicity are combined at a certain rate, hydrophilic segment and hydrophobic segment are associated with solute permeability and mechanical strength, respectively. 

Platelet-neutrophil complex formation is enhanced by the activations of both platelet and complement which are triggered when hemodialysis membrane contacts with blood. Neutrophil activation, such as radical oxygen production, is triggered by platelet-neutrophil complex [8]. Since these activations of leukocyte and platelet affect hemorheology, microcirculatory disorder could be triggered during HD. It has been reported that impact on leukocyte and platelet is lower in EVAL membrane compared with that in PS membrane or cellulose diacetate membrane [9]. It has also been reported that microcirculatory disorder is hard to occur during HD with EVAL membrane due to its excellent biocompatibility [10]. 

### 3.4. Vitamin E-Coated Polysulfone (PS) Membrane

During hemodialysis session, large amount of antioxidant materials is consumed in the body since the neutrophilic activation, the radical oxygen production, and the resulting oxidant stress reach excess states by the blood contact with hemodialysis membrane [11]. In order to reduce these excess states of the oxidant stress, following dialysis membrane has been developed that vitamin E with antioxidant effect is coated on cellulose membrane or PS membrane [12], and it has been reported that active oxygen production is suppressed and that oxidant stress is reduced during hemodialysis [13, 14]. 

## 4. Other Blood Purification Devices

In Japan, a variety of adsorption materials, which more selectively remove disease-related substances based on principle of adsorption, have been developed and applied clinically. Adsorption materials used for blood purification are roughly divided into blood adsorbent (hemoadsorption column) for direct hemoperfusion and plasma adsorbent (plasma adsorption column) for plasma perfusion. 

### 4.1. Hemoadsorption Column

#### 4.1.1. *β*2-MG Adsorption Column (Lixelle)

**Figure 7:**
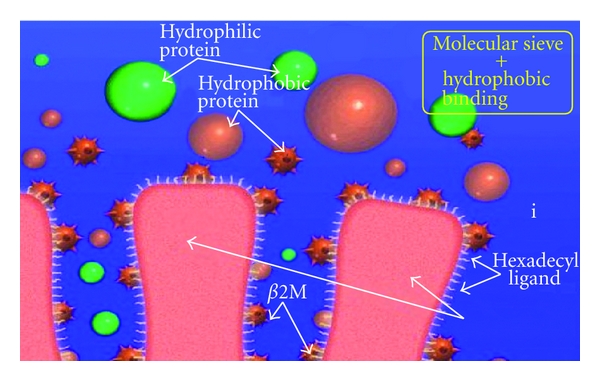
The schema of the Lixelle column. The Lixelle column is designed to adsorb *β*2-MG selectively by combined use of hydrophobic interaction and adequate pore size.

*β*2-MG, which is a hydrophobic protein with molecular weight of 11,800, is accumulated during long-term dialysis. Further, insoluble amyloid protein which consists primarily of *β*2-MG with various modifications is accumulated in organs throughout the body and it results in a development of amyloidosis (dialysis-related amyloid (DRA)). DRA causes deterioration in ADL and QOL of dialysis patients. Accumulation of *β*2-MG not only leads ADL and QOL of dialysis patients to deteriorate but also adversely affects survival. Therefore, HD or HDF based on high-flux membrane has been performed aiming at removing *β*2-MG from the body, and ultrapure dialysate and excellent biocompatible dialyzers have been applied aiming at inhibiting *β*2-MG production. Direct hemoperfusion with *β*2-MG adsorber column (Lixelle: Kaneka Co., Ltd., Osaka, Japan) was developed in 1996 which is capable of powerfully removing *β*2-MG from the blood [15]. The Lixelle column contains 350 mL of porous cellulose adsorbent beads with a diameter of about 460 *μ*m, in which a hexadecyl group with high hydrophobicity is used as ligand porous cellulose adsorbent beads. It incorporates peptides and proteins with a molecular weight of 4,000 to 20,000 dalton by the molecule sieving effect of the surface pores of the beads (Figure 7), whereas total protein, albumin, immunoglobulins, and lipids are not adsorbed [16]. Through the clinical effect of Lixelle, improvements not only in arthralgia, nocturnal awakening, and ADL but also in pinch strength and median motor terminal latency were observed after a year of Lixelle therapy [17, 18]. In Lixelle, biocompatibility is a crucial issue since blood is directly perfused through adsorbent. As it has been reported that HD combined with Lixelle increases myeloperoxidase and polymorphonuclear leukocyte-elastase compared with HD not combined with it, further examinations of the biocompatibility are required [19].

#### 4.1.2. Endotoxin Removal Column (Toraymyxin)

**Figure 8:**
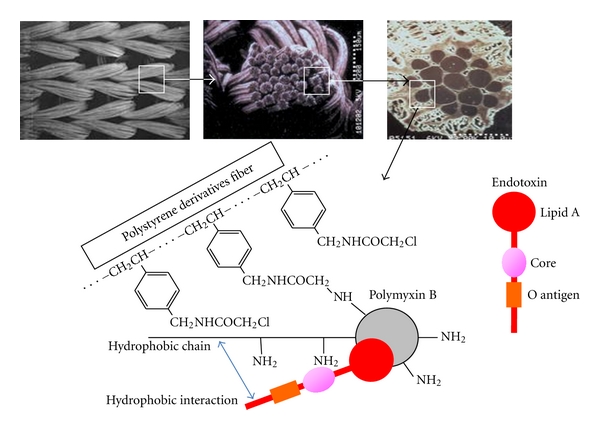
The schema of toraymyxin. Toraymyxin is filled with polymyxin-B-immobilized polystyrene derivative fibers. Polymyxin B fixed on a fibrous carrier exerts a function to neutralize the activity of endotoxin by combining with lipid A, which is an active center of the activity.

A column for directly removing endotoxin from the blood (Toraymyxin: Toray Industries, Osaka, Japan) was developed for treatment of endotoxin shock which is one of the causes of prerenal acute kidney injury [20]. Toraymyxin is filled with polymyxin-B-immobilized polystyrene derivative fibers. Polymyxin B fixed on a fibrous carrier exerts a function to neutralize the activity of endotoxin by combining with lipid A, which is an active center of the activity (Figure 8). A new functional mechanism has been reported for Toraymyxin that it has an effect to adsorb endogenous cannabinoid which is an early mediator of endotoxin shock [21]. In patients with septic shock, a lot of clinical effects of Toraymyxin have been reported, including elevation in blood pressure, reduction of vasopressor, decrease in 28-day mortality and hospital mortality, and improvement in organ failure [22]. 

 Even though a risk that a possibility of fixed polymyxin B is eluted cannot be totally excluded, clinically apparent side effect has not been recognized [23]. 

#### 4.1.3. Leukocyte Removal Therapy (LRT)

LRT is a therapy to directly remove leukocyte from the blood which is considered to be related as a cause of disease or symptoms. There are two types of therapy for the present, that is, leukocytapheresis (LCAP) and granulocytapheresis (GCAP), and they have clinical indications for inflammatory bowel disease and rheumatoid arthritis in Japan [24–27].

**(1) LCAP**

Polyethylene terephthalate (PET) fiber with a diameter of 3 *μ*m or less has a property to adsorb almost 100% of granulocyte and monocyte and 30 to 60% of lymphocyte and platelet. Cellsorba (Asahi Medical Co., Tokyo, Japan) is comprised of a rolled nonwoven fabric of PET fiber with a fiber diameter ranging from 0.8 to 2.8 *μ*m for selectively removing leukocyte.

**Figure 9:**
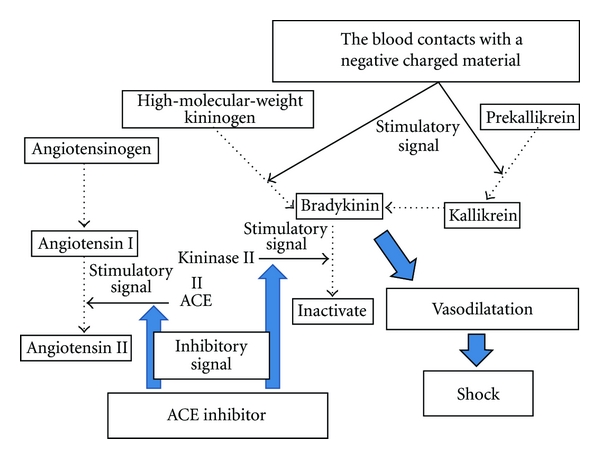
The mechanism of bradykinin-induced anaphylactic shock. When the blood contacts with a negatively charged material, it results in production of Bradykinin (BK) having vasodilating action. The bioactivity of this BK is inactivated by kininase II which is an enzyme identical to ACE. Therefore, the function of kininase II which is an enzyme identical to ACE is inhibited by taking ACE inhibitor and it causes increase in BK concentration in the blood, vasodilatation, and shock symptom being unable to inactivate BK.

Problems of biocompatibility on PET include bradykinin shock which occurs in patients who are orally taking angiotensin-converting enzyme (ACE) inhibitor since PET is negatively charged. Blood coagulation system is strongly activated by the blood contacts with a negatively charged material, and it results in production of bradykinin (BK) having vasodilating action. This BK is inactivated by kininase II which is an enzyme identical to ACE. Therefore, the function of kininase II which is an enzyme identical to ACE is inhibited by taking ACE inhibitor and it causes increase in BK concentration in the blood, vasodilatation, and shock symptom (Figure 9). Besides, some reactions on the skin of blood return, such as pain, redness, and swelling, could be brought by various chemical mediators which are released from active leukocyte and platelet adsorbed within Cellsorba.

**(2) GCAP**

Cellulose acetate (CA) beads combine with immunoglobulin (IgG) and complement active C3b/C3bi. Granulocyte and monocyte have receptors for Fc of IgG or complement and are selectively trapped via immunoglobulin and complement which are combined with CA and these receptors.

Adacolumn (JIMRO, Takasaki, Japan) is a purifier filled with 30,000 of CA beads with a diameter of 3 mm for selectively removing granulocyte and monocyte. During the purification, neither lymphocyte nor platelet is removed. The complications, which included venous pressure elevation, venous access difficulty, coagulation in blood circuit, and difficulty in returning blood, occurred during 2.3% of GCAP. However, there were no serious adverse events [28].

### 4.2. Plasma Adsorption Column

#### 4.2.1. Plasma Adsorption Based on a Principle of Hydrophobic Bonding (Immusorba PH-350/TR-350)

**Figure 10:**
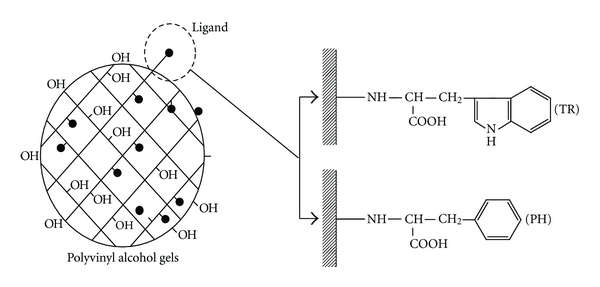
The structural formulas of Immusorba PH-350 and TR-350. These columns covalently bond with the hydroxyl group on the surface of porous polyvinyl alcohol gel with the amino group of amino acid. Immusorba PH-350 is using phenylalanine as ligand and TR-350 is using tryptophan.

**Figure 11:**
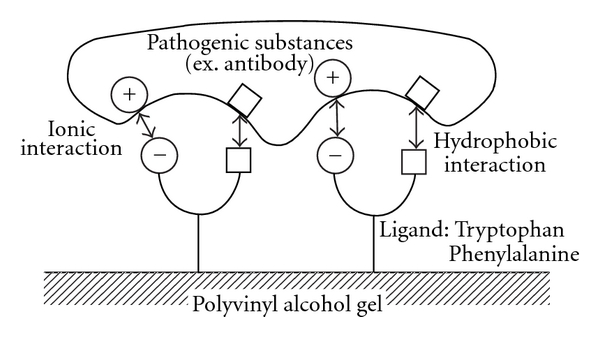
The schema of Immusorba PH-350 and TR-350. The affinity between ligand of these columns and antibody was mainly hydrophobic interaction and partially caused by the ionic interaction based on carboxylic group.

Autoantibody and immune complex have hydrophobic group. Both phenylalanine and tryptophan are hydrophobic amino acids widely distributed in the body. Immusorba PH-350 (Asahi Medical Co., Tokyo, Japan) is using phenylalanine as a ligand, and TR-350 (Asahi Medical Co., Tokyo, Japan) is using tryptophan as a ligand. These columns covalently bond with the hydroxyl group on the surface of porous polyvinyl alcohol gel with the amino group of amino acid (Figure 10). These columns could selectively remove autoantibody or immune complex by hydrophobic bonding. Further, as these adsorbing materials are charged negatively, the affinity between ligand of these columns and antibody was mainly hydrophobic interaction and was partially caused by the ionic interaction based on carboxylic group (Figure 11). The clinical effects of these columns have been reported on lupus nephritis, myasthenia gravis, multiple sclerosis, and other collagen diseases [29–33].

**Figure 12:**
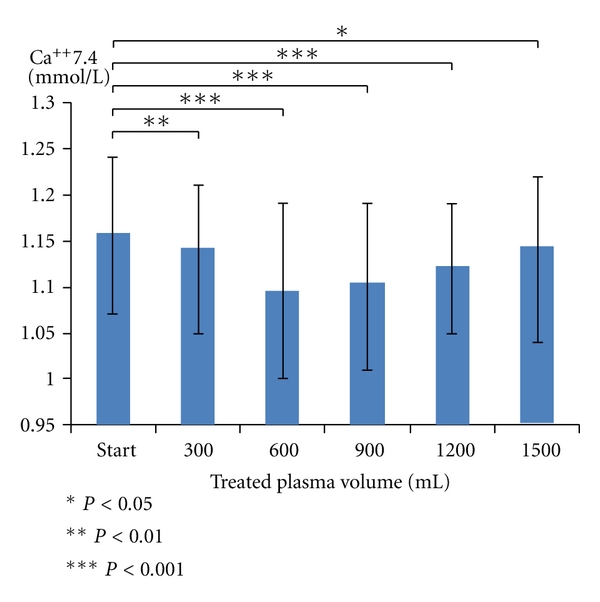
The change of serum Ca ion level during immunoadsorption with Immusorba TR-350. Reduction of Ca ion level could be observed during treatment by using Immusorba TR-350, because positive ion could be absorbed by the negative charge.

As both Immusorba PH-350 and TR-350 are charged negatively, shock symptom is triggered by bradykinin in patients who are taking ACE inhibitor. In addition, reduction in Ca ion level could be observed temporarily during treatment (Figure 12) because the positive ion could be absorbed by the negative charge. 

#### 4.2.2. Plasma Adsorption Based on Electrostatic Bond (Liposorber, Selesorb)

**Figure 13:**
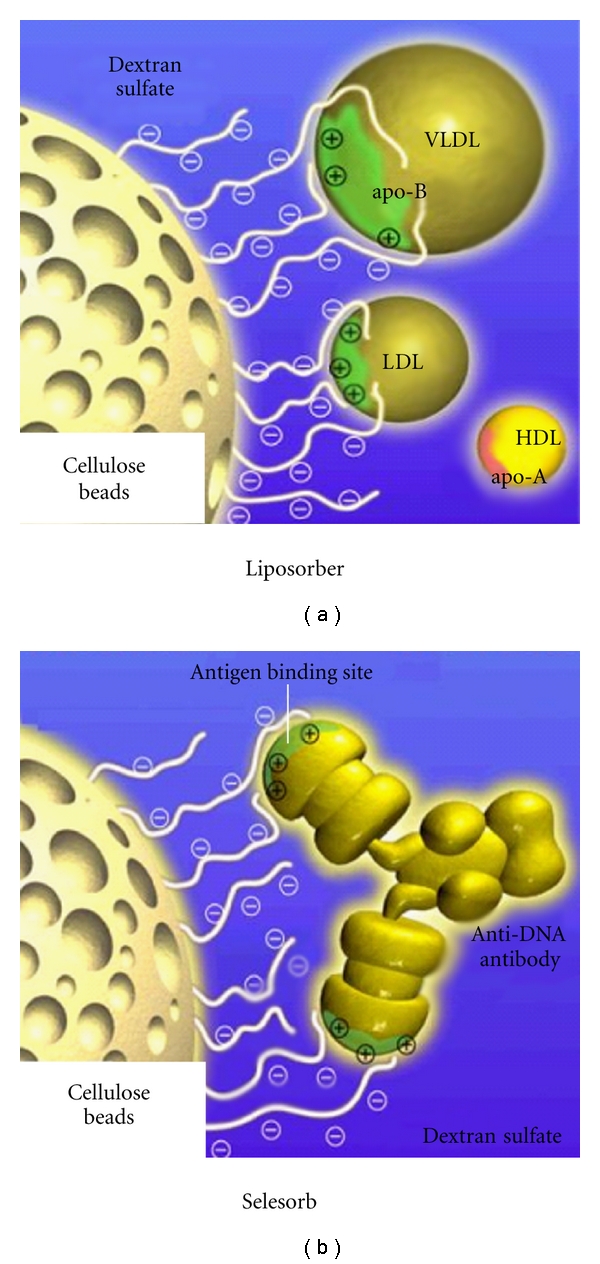
The schema of the Liposorber and Selesorb. Apo-B, anti-DNA antibody, immune complex, or antigen-recognizing site on anticardiolipin antibody is charged with positive charge. Liposorber and Selesorb include cellulose beads with the negative charge of dextran sulphate. The affinity between negative charge of these columns and these positive charged etiological substances was mainly caused by the ionic interaction.

**Figure 14:**
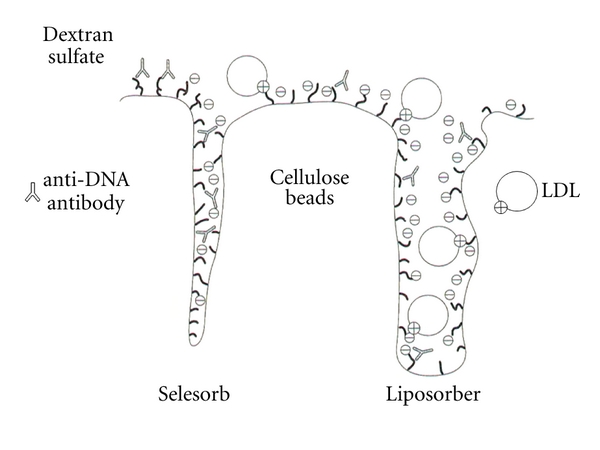
The difference of between Liposorber and Selesorb. The pore size of Selesorb beads is smaller than that of Liposorber so that causative antibodies including anti-DNA antibody are selectively adsorbed avoiding larger-size molecules such as LDL.

Apo-B, anti-DNA antibody, immune complex, or antigen-recognizing site on anticardiolipin antibody is charged positively. Liposorber (Kaneka Co., Ltd., Osaka, Japan) and Selesorb (Kaneka Co., Ltd., Osaka, Japan) are a column, which includes cellulose beads with the negative charge of dextran sulphate. The affinity between negative charge of these columns and these positive-charged etiological substances was mainly caused by the ionic interaction (Figure 13). Alternatively, Selesorb is devised to enhance selectivity of anti-DNA antibody, immune complex, and anticardiolipin antibody which are smaller than apo-B by making pore structure of cellulose gel smaller (Figure 14). As both Liposorber and Selesorb are charged negatively, they have a risk of developing shock symptom by bradykinin in patients who are taking ACE inhibitor.

**(1) Liposorber**

LDL cholesterol level is drastically reduced by 60 to 80% by single LDL apheresis using Liposorber, whereas HDL cholesterol level is hardly reduced. LDL apheresis using Liposorber is conducted for the heterofamilial hyperlipidemic patients, and clinical effects have been reported that incidence of cardiovascular event reduced by 70% compared with those in groups treated based on medication alone [34]. Further, the clinical effects of LDL apheresis using Liposorber have been reported also on arteriosclerosis obliterans for which hyperlipidemia is one of the risk factors [35]. The mechanisms are supposed to have impacts on (i) improvement in viscosity of blood and plasma, (ii) improvement in erythrocyte deformability, (iii) increase of vasodilator such as bradykinin and NO, (ix) reduction in coagulation factor such as fibrinogen, (x) reduction in cell adhesion factor, (xi) inhibition of platelet activation, and (xii) inhibition of oxidizability of LDL. Moreover, the progression of renal dysfunction due to focal glomerulosclerosis (FGS) which presents refractory nephrotic syndrome to steroids was ameliorated by LDL apheresis. Glomerular sclerosis is thought to be caused by systemic or intraglomerular hypertension associated with arteriosclerosis progress, enhanced cytokine secretion as well as mesangial cell proliferation associated with increase of macrophage in glomerulus, and disorder of mesangial cell, and they are associated with increase of LDL in the blood [36].

In a multi-institutional study of LDL apheresis using Liposorber for steroid-resistant nephrotic syndrome, significant reduction in urine protein and elevation in serum albumin level were recognized which shortened the days required for reducing urine protein level to less than 3.5 g/day. In addition, the following clinical effects have been also reported that remission rate in two years after completion of treatment is significantly high [37]. This paper pointed out the following factors for possible mechanisms of LDL apheresis using Liposorber: improvement of hyperlipidemia as well as inhibition of hypercoagulation, correction of disorder of renal lipid mediator (thromboxane A2 hyperproduction state), inhibition of infiltration of macrophage to glomerulus based on inhibitory action on cytokine production, restoration of steroid-responsiveness, and enhancement of intracellular incorporation of cocurrent use of cyclosporine.

**(2) Selesorb**

By inmmunoabsorption therapy with Selesorb, the mean removal rate of anti-DNA antibody was 34%, moreover, anticardiolipin antibody and immune complex were efficiently removed. The clinical features of systemic lupus erythematosus, such as facial erythema and hematologic disorder, were also improved without severe adverse effects [38].

## 5. Conclusion

It is important that blood purification therapy should be performed consistently evaluating not only risks associated with these bioincompatibilities but also loads to cardiovascular system posed by extracorporeal circulation, risks relating to vascular access creation, and exacerbation of hemorrhagic tendency caused by anticoagulant, together with advantages obtained from treatments. The characteristics of biocompatibility of each material mentioned here will bring important information for safer blood purification therapy.
